# *trans*-Tetra­aqua­bis­[2-(4-chloro­phen­oxy)acetato-κ*O* ^1^]nickel(II)

**DOI:** 10.1107/S1600536812045540

**Published:** 2012-11-10

**Authors:** Jamshid Ashurov, Mavlonbek Ziyaev, Bakhtiyar Ibragimov, Samat Talipov

**Affiliations:** a Institute of Bioorganic Chemistry, Academy of Sciences of Uzbekistan, M. Ulugbek Str. 83, Tashkent, 700125 Uzbekistan

## Abstract

In the title compound, [Ni(C_8_H_6_ClO_3_)_2_(H_2_O)_4_], the Ni^II^ ion is located on a crystallographic inversion centre and is octa­hedrally coordinated by two 2-(4-chloro­phen­oxy)acetate ligands in axial positions and by four water mol­ecules in the equatorial plane. The acetate ligands are bound to the Ni^II^ ion in a monodentate manner through a carboxyl­ate O atom. In the crystal, O—H⋯O hydrogen bonds link the mol­ecules, forming a two-dimensional supra­molecular network lying parallel to the *ab* plane.

## Related literature

For inter­actions of metal ions with amino acids, see: Daniele *et al.* (2008 ▶); Parkin (2004 ▶). For the crystal structures of related 4-chloro­phen­oxy­acetate complexes, see: Liwporncharoenvong & Luck (2005 ▶); Smith *et al.* (1980 ▶); Wang *et al.* (2008 ▶).
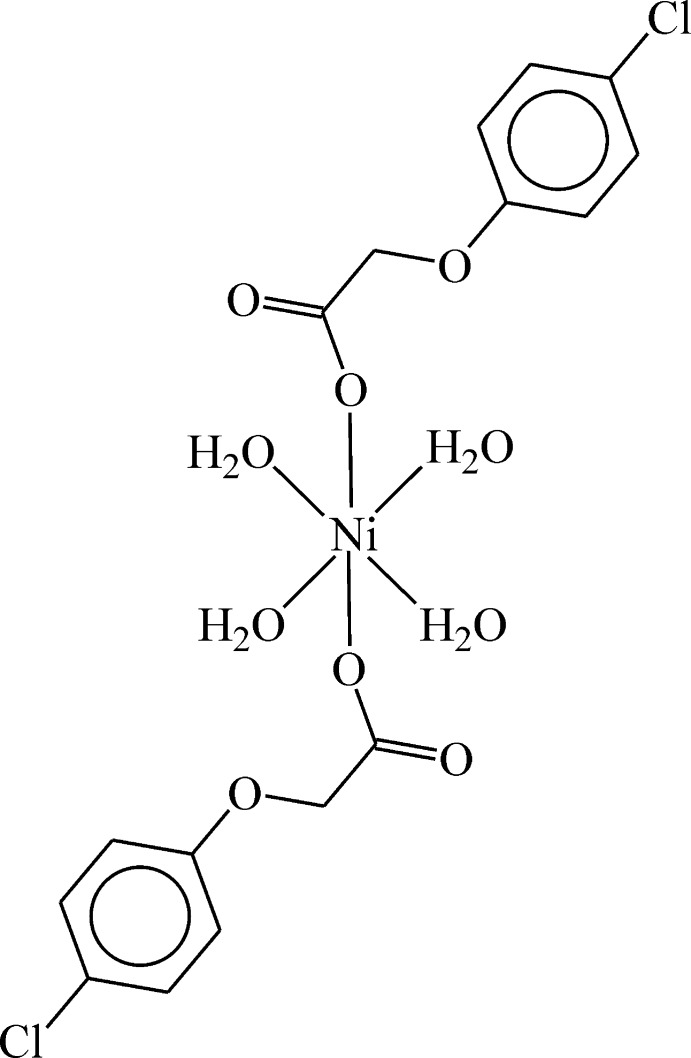


## Experimental

### 

#### Crystal data

[Ni(C_8_H_6_ClO_3_)_2_(H_2_O)_4_]
*M* *_r_* = 501.93
Triclinic, 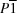

*a* = 4.894 (3) Å
*b* = 5.769 (4) Å
*c* = 18.369 (9) Å
α = 97.226 (3)°
β = 90.088 (4)°
γ = 96.796 (4)°
*V* = 510.8 (5) Å^3^
*Z* = 1
Cu *K*α radiation
μ = 4.25 mm^−1^
*T* = 293 K
0.45 × 0.22 × 0.2 mm

#### Data collection

Oxford Diffraction Xcalibur Ruby diffractometer
Absorption correction: multi-scan (*CrysAlis PRO*; Oxford Diffraction, 2009 ▶) *T*_min_ = 0.226, *T*_max_ = 1.000
4607 measured reflections
2059 independent reflections
1759 reflections with *I* > 2σ(*I*)
*R*_int_ = 0.052

#### Refinement

*R*[*F* ^2^ > 2σ(*F* ^2^)] = 0.099
*wR*(*F* ^2^) = 0.272
*S* = 1.13
2059 reflections
135 parameters
H-atom parameters constrained
Δρ_max_ = 2.78 e Å^−3^
Δρ_min_ = −1.32 e Å^−3^

### 

Data collection: *CrysAlis PRO* (Oxford Diffraction, 2009 ▶); cell refinement: *CrysAlis PRO*; data reduction: *CrysAlis PRO*; program(s) used to solve structure: *SHELXS97* (Sheldrick, 2008 ▶); program(s) used to refine structure: *SHELXL97* (Sheldrick, 2008 ▶); molecular graphics: *XP* in *SHELXTL* (Sheldrick, 2008 ▶) and *PLATON* (Spek, 2009 ▶); software used to prepare material for publication: *SHELXL97*.

## Supplementary Material

Crystal structure: contains datablock(s) I, global. DOI: 10.1107/S1600536812045540/su2512sup1.cif

Structure factors: contains datablock(s) I. DOI: 10.1107/S1600536812045540/su2512Isup2.hkl

Additional supplementary materials:  crystallographic information; 3D view; checkCIF report

## Figures and Tables

**Table 1:** Hydrogen-bond geometry (Å, °)

*D*—H⋯*A*	*D*—H	H⋯*A*	*D*⋯*A*	*D*—H⋯*A*
O2*W*—H2*WA*⋯O3	0.87	1.93	2.679 (4)	144
O2*W*—H2*WB*⋯O1*W* ^i^	0.87	2.01	2.843 (4)	162
O1*W*—H1*WA*⋯O3^ii^	0.86	1.98	2.732 (4)	145
O1*W*—H1*WB*⋯O1^iii^	0.86	2.21	2.978 (4)	148
O1*W*—H1*WB*⋯O2^iii^	0.86	2.18	2.861 (4)	135

Symmetry codes: (i) 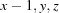; (ii) 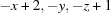; (iii) 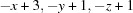.

**Fig. 1.**
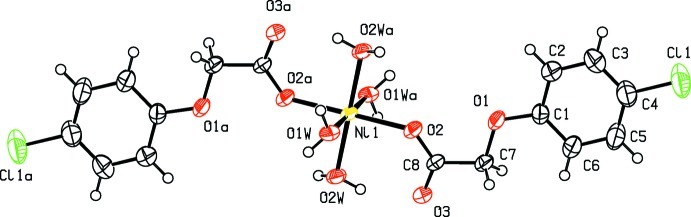
The molecular structure of the title compound with the atom-labeling scheme. Displacement ellipsoids are drawn at the 30% probability level [symmetry code: (a) -x+2, -y+1, -z+1].

**Fig. 2.**
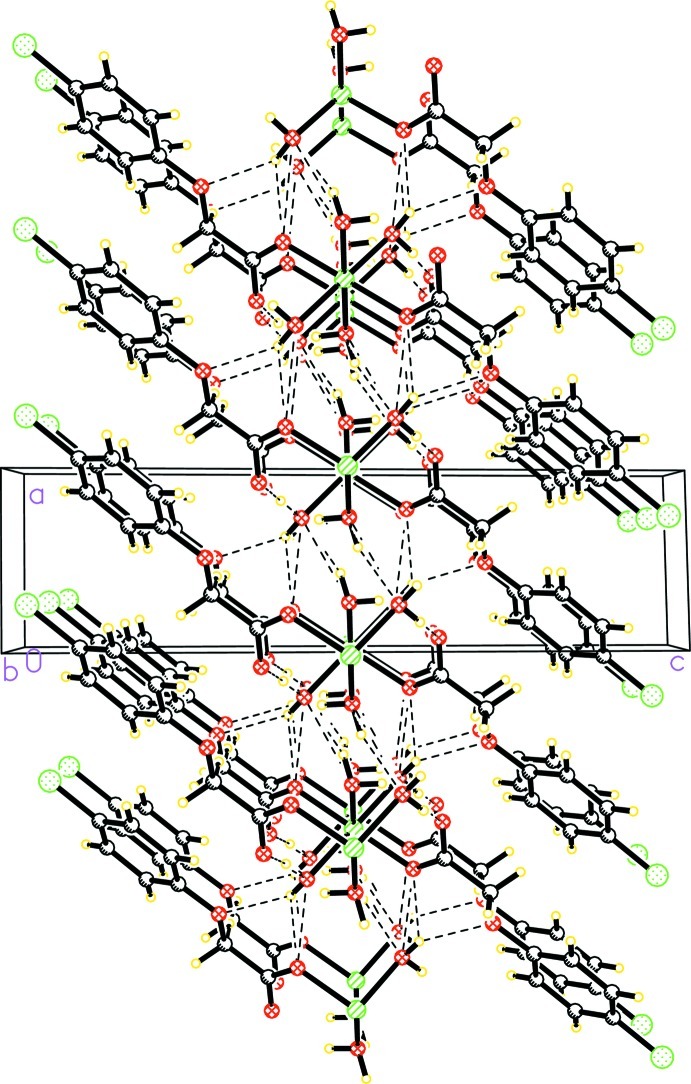
A view along the a axis of the crystal packing of title compound. Hydrogen bonds are shown as dashed lines - see Table 1 for details.

## supplementary crystallographic information

### Comment

The interaction of transition metal ions with biologically active molecules such as amino acids and various organic acids is very important in biological systems (Parkin, 2004; Daniele *et al.*, 2008). 4-chlorophenoxyacetic acid is one such acid that has been used to form various complexes with transition metals (Liwporncharoenvong & Luck, 2005; Smith *et al.*, 1980; Wang *et al.*, 2008). We report herein on the synthesis and crystal structure of a new nickel(II) complex involving this ligand.

In the title compound the Ni^II^ ion is located on a crystallographic inversion centre (Fig. 1). The Ni^II^ ion is octahedral coordinated by two *p*-chlorophenoxyacetato ligands in axial positions [Ni—O = 2.060 (3) Å] and by four water molecules in the equatorial plane [Ni—O = 2.084 (3) and 2.039 (3) Å]. The *p*-chlorophenoxyacetato ligands are bound to the Ni^II^ ion in a monodentate manner through a carboxylate O atom.

In the crystal, molecules are linked via O-H···O hydrogen bonds (Table 1), resulting in the formation of a complex two-dimensional supramolecular network lying parallel to the ab plane (Fig. 2).

### Experimental

A solution of 4-chlorophenoxyacetic acid (40 mg, 0.2 mmol) in ethanol (3 ml) was added to a solution of Ni(CH_3_OO)_2_ [15.96 mg 0.1 mmol] in water (1.5 ml) and stirred for 10 min at 303 K. Slow evaporation of the resulting solution gave green block-like crystals of the title compound suitable for X-ray analysis.

### Refinement

All the H atoms were included in calculated positions and treated as riding: C—H = 0.97 Å (methylene), 0.93 Å (aromatic) and O—H = 0.86 Å, with U_iso_(H) = k × U_eq_(parent atom), where k = 1.5 for water H atoms and = 1.2 for other H atoms. In the final difference Fourier map the highest residual density peak is located near the metal atom.

### Crystal data

**Table:**

[Ni(C_8_H_6_ClO_3_)_2_(H_2_O)_4_]	*V* = 510.8 (5) Å^3^
*M**_r_* = 501.93	*Z* = 1
Triclinic, *P*1	*F*(000) = 258
Hall symbol: -P 1	*D*_x_ = 1.632 Mg m^−^^3^
*a* = 4.894 (3) Å	Cu *K*α radiation, λ = 1.54184 Å
*b* = 5.769 (4) Å	µ = 4.25 mm^−^^1^
*c* = 18.369 (9) Å	*T* = 293 K
α = 97.226 (3)°	Block, green
β = 90.088 (4)°	0.45 × 0.22 × 0.2 mm
γ = 96.796 (4)°	

### Data collection

**Table:**

Oxford Diffraction Xcalibur Ruby diffractometer	2059 independent reflections
Radiation source: fine-focus sealed tube	1759 reflections with *I* > 2σ(*I*)
Graphite monochromator	*R*_int_ = 0.052
Detector resolution: 10.2576 pixels mm^-1^	θ_max_ = 75.9°, θ_min_ = 4.9°
ω scans	*h* = −5→6
Absorption correction: multi-scan (*CrysAlis PRO*; Oxford Diffraction, 2009)	*k* = −7→6
*T*_min_ = 0.226, *T*_max_ = 1.000	*l* = −19→23
4607 measured reflections	

### Refinement

**Table:**

Refinement on *F*^2^	Primary atom site location: structure-invariant direct methods
Least-squares matrix: full	Secondary atom site location: difference Fourier map
*R*[*F*^2^ > 2σ(*F*^2^)] = 0.099	Hydrogen site location: inferred from neighbouring sites
*wR*(*F*^2^) = 0.272	H-atom parameters constrained
*S* = 1.13	*w* = 1/[σ^2^(*F*_o_^2^) + (0.2*P*)^2^] where *P* = (*F*_o_^2^ + 2*F*_c_^2^)/3
2059 reflections	(Δ/σ)_max_ = 0.002
135 parameters	Δρ_max_ = 2.78 e Å^−^^3^
0 restraints	Δρ_min_ = −1.32 e Å^−^^3^

### Special details

**Table:**

Geometry. All e.s.d.'s (except the e.s.d. in the dihedral angle between two l.s. planes) are estimated using the full covariance matrix. The cell e.s.d.'s are taken into account individually in the estimation of e.s.d.'s in distances, angles and torsion angles; correlations between e.s.d.'s in cell parameters are only used when they are defined by crystal symmetry. An approximate (isotropic) treatment of cell e.s.d.'s is used for estimating e.s.d.'s involving l.s. planes.
Refinement. Refinement of *F*^2^ against ALL reflections. The weighted *R*-factor *wR* and goodness of fit *S* are based on *F*^2^, conventional *R*-factors *R* are based on *F*, with *F* set to zero for negative *F*^2^. The threshold expression of *F*^2^ > σ(*F*^2^) is used only for calculating *R*-factors(gt) *etc*. and is not relevant to the choice of reflections for refinement. *R*-factors based on *F*^2^ are statistically about twice as large as those based on *F*, and *R*- factors based on ALL data will be even larger.

### Fractional atomic coordinates and isotropic or equivalent isotropic displacement parameters (Å^2^)

**Table:**

	*x*	*y*	*z*	*U*_iso_*/*U*_eq_	
Ni1	1.0000	0.5000	0.5000	0.0282 (4)	
Cl1	2.2639 (4)	0.3457 (4)	0.04767 (9)	0.0837 (7)	
O2W	0.7333 (6)	0.2008 (5)	0.50078 (16)	0.0359 (7)	
H2WA	0.7214	0.1192	0.4577	0.054*	
H2WB	0.5706	0.2354	0.5131	0.054*	
O1	1.5117 (7)	0.3222 (6)	0.29609 (16)	0.0413 (8)	
O1W	1.2630 (6)	0.3692 (5)	0.56991 (15)	0.0344 (7)	
H1WA	1.1689	0.2886	0.5999	0.052*	
H1WB	1.3660	0.4834	0.5951	0.052*	
O3	0.9237 (6)	0.0301 (5)	0.37074 (15)	0.0379 (7)	
O2	1.2116 (7)	0.3526 (5)	0.41255 (16)	0.0369 (7)	
C1	1.6813 (9)	0.3129 (8)	0.2362 (2)	0.0383 (10)	
C7	1.3174 (9)	0.1227 (7)	0.3018 (2)	0.0358 (9)	
H7A	1.4128	−0.0122	0.3075	0.043*	
H7B	1.2049	0.0863	0.2574	0.043*	
C8	1.1359 (8)	0.1725 (6)	0.36740 (19)	0.0293 (8)	
C2	1.8536 (13)	0.5167 (10)	0.2301 (3)	0.0539 (13)	
H2	1.8489	0.6472	0.2651	0.065*	
C6	1.6845 (11)	0.1169 (10)	0.1845 (3)	0.0491 (12)	
H6	1.5671	−0.0199	0.1885	0.059*	
C5	1.8678 (12)	0.1279 (12)	0.1261 (3)	0.0564 (13)	
H5	1.8740	−0.0029	0.0912	0.068*	
C4	2.0382 (12)	0.3318 (12)	0.1203 (3)	0.0556 (13)	
C3	2.0336 (14)	0.5284 (12)	0.1721 (3)	0.0621 (15)	
H3	2.1495	0.6659	0.1680	0.075*	

### Atomic displacement parameters (Å^2^)

**Table:**

	*U*^11^	*U*^22^	*U*^33^	*U*^12^	*U*^13^	*U*^23^
Ni1	0.0331 (6)	0.0178 (6)	0.0305 (6)	−0.0016 (4)	0.0044 (4)	−0.0044 (4)
Cl1	0.0815 (12)	0.1170 (16)	0.0516 (9)	0.0067 (10)	0.0316 (8)	0.0111 (9)
O2W	0.0349 (15)	0.0225 (14)	0.0465 (16)	−0.0025 (11)	0.0093 (12)	−0.0043 (11)
O1	0.0472 (18)	0.0345 (16)	0.0357 (15)	−0.0041 (13)	0.0120 (13)	−0.0122 (12)
O1W	0.0383 (16)	0.0235 (13)	0.0385 (14)	−0.0042 (11)	−0.0019 (11)	−0.0007 (10)
O3	0.0471 (18)	0.0272 (15)	0.0356 (14)	−0.0039 (12)	0.0041 (12)	−0.0025 (11)
O2	0.0440 (17)	0.0270 (15)	0.0350 (14)	−0.0025 (12)	0.0047 (12)	−0.0085 (11)
C1	0.040 (2)	0.041 (2)	0.0317 (18)	0.0007 (18)	0.0084 (16)	−0.0002 (16)
C7	0.040 (2)	0.028 (2)	0.0350 (19)	−0.0020 (16)	0.0032 (16)	−0.0069 (15)
C8	0.035 (2)	0.0217 (18)	0.0299 (17)	0.0043 (14)	−0.0009 (14)	−0.0035 (13)
C2	0.067 (4)	0.041 (3)	0.047 (3)	−0.008 (2)	0.014 (2)	−0.004 (2)
C6	0.057 (3)	0.046 (3)	0.039 (2)	0.000 (2)	0.010 (2)	−0.0071 (19)
C5	0.058 (3)	0.067 (4)	0.040 (2)	0.006 (3)	0.011 (2)	−0.010 (2)
C4	0.056 (3)	0.068 (4)	0.042 (2)	0.001 (3)	0.010 (2)	0.008 (2)
C3	0.068 (4)	0.060 (3)	0.055 (3)	−0.009 (3)	0.021 (3)	0.009 (3)

### Geometric parameters (Å, º)

**Table:**

Ni1—O2W^i^	2.039 (3)	O2—C8	1.263 (5)
Ni1—O2W	2.039 (3)	C1—C2	1.380 (7)
Ni1—O2	2.060 (3)	C1—C6	1.383 (7)
Ni1—O2^i^	2.060 (3)	C7—C8	1.516 (5)
Ni1—O1W^i^	2.084 (3)	C7—H7A	0.9700
Ni1—O1W	2.084 (3)	C7—H7B	0.9700
Cl1—C4	1.737 (5)	C2—C3	1.386 (7)
O2W—H2WA	0.8668	C2—H2	0.9300
O2W—H2WB	0.8667	C6—C5	1.403 (7)
O1—C1	1.379 (5)	C6—H6	0.9300
O1—C7	1.419 (5)	C5—C4	1.375 (9)
O1W—H1WA	0.8641	C5—H5	0.9300
O1W—H1WB	0.8642	C4—C3	1.388 (9)
O3—C8	1.252 (5)	C3—H3	0.9300
			
O2W^i^—Ni1—O2W	180.0	C2—C1—C6	120.6 (4)
O2W^i^—Ni1—O2	87.58 (12)	O1—C7—C8	109.7 (3)
O2W—Ni1—O2	92.42 (12)	O1—C7—H7A	109.7
O2W^i^—Ni1—O2^i^	92.42 (12)	C8—C7—H7A	109.7
O2W—Ni1—O2^i^	87.58 (12)	O1—C7—H7B	109.7
O2—Ni1—O2^i^	180.00 (11)	C8—C7—H7B	109.7
O2W^i^—Ni1—O1W^i^	89.06 (12)	H7A—C7—H7B	108.2
O2W—Ni1—O1W^i^	90.94 (13)	O3—C8—O2	126.7 (4)
O2—Ni1—O1W^i^	91.59 (13)	O3—C8—C7	116.0 (3)
O2^i^—Ni1—O1W^i^	88.41 (13)	O2—C8—C7	117.3 (4)
O2W^i^—Ni1—O1W	90.95 (13)	C1—C2—C3	120.6 (5)
O2W—Ni1—O1W	89.05 (12)	C1—C2—H2	119.7
O2—Ni1—O1W	88.41 (13)	C3—C2—H2	119.7
O2^i^—Ni1—O1W	91.59 (13)	C1—C6—C5	118.9 (5)
O1W^i^—Ni1—O1W	180.00 (12)	C1—C6—H6	120.6
Ni1—O2W—H2WA	110.5	C5—C6—H6	120.6
Ni1—O2W—H2WB	110.4	C4—C5—C6	120.1 (5)
H2WA—O2W—H2WB	108.4	C4—C5—H5	120.0
C1—O1—C7	117.3 (3)	C6—C5—H5	120.0
Ni1—O1W—H1WA	110.3	C5—C4—C3	120.8 (5)
Ni1—O1W—H1WB	110.4	C5—C4—Cl1	120.1 (5)
H1WA—O1W—H1WB	108.6	C3—C4—Cl1	119.1 (4)
C8—O2—Ni1	128.6 (3)	C2—C3—C4	119.0 (5)
O1—C1—C2	115.3 (4)	C2—C3—H3	120.5
O1—C1—C6	124.0 (4)	C4—C3—H3	120.5

Symmetry code: (i) −*x*+2, −*y*+1, −*z*+1.

### Hydrogen-bond geometry (Å, º)

**Table:**

*D*—H···*A*	*D*—H	H···*A*	*D*···*A*	*D*—H···*A*
O2*W*—H2*WA*···O3	0.87	1.93	2.679 (4)	144
O2*W*—H2*WB*···O1*W*^ii^	0.87	2.01	2.843 (4)	162
O1*W*—H1*WA*···O3^iii^	0.86	1.98	2.732 (4)	145
O1*W*—H1*WB*···O1^iv^	0.86	2.21	2.978 (4)	148
O1*W*—H1*WB*···O2^iv^	0.86	2.18	2.861 (4)	135

Symmetry codes: (ii) *x*−1, *y*, *z*; (iii) −*x*+2, −*y*, −*z*+1; (iv) −*x*+3, −*y*+1, −*z*+1.
